# Supplementary material for: Triterpenes and Phenolic Compounds from Euphorbia deightonii with Antiviral Activity against Herpes Simplex Virus Type-2
Source: Plants (Basel). 2022 Mar 13;11(6):764. doi: 10.3390/plants11060764 (PMC8955370; doi:10.3390/plants11060764)
Supplement: Supplementary file 1 [file plants-11-00764-s001.zip › plants-1635912-supplementary.pdf]

Triterpenes and Phenolic Compounds from *Euphorbia deightonii* with Antiviral Activity against Herpes Simplex Virus Type 2

SUPPLEMENTARY MATERIAL

Muhammad Bello Saidu,[1] Norbert Kúsz,[1] Yu-Chi Tsai,[1] Máté Vágvölgyi,[1] Róbert Berkecz,[3] Dávid Kókai,[3] Katalin Burián,[3] Judit Hohmann,[1,4] Dóra Rédei[1]*

[1] Department of Pharmacognosy, University of Szeged, 6720 Szeged, Hungary; bello.saidu@pharmacognosy.hu; kusznorbert@gmail.com; hohmann.judit@szte.hu; redei@pharmacognosy.hu

[2] Albert Szent-Györgyi Medical School, Department of Medical Microbiology, University of Szeged, Dóm tér 10, 6720 Szeged, Hungary; kokai.david@med.u-szeged.hu; burian.katalin@med.u-szeged.hu

[3] Institute of Pharmaceutical Analysis, University of Szeged, Somogyi u. 4, 6720 Szeged, Hungary; berkecz.robert@szte.hu

[4] Interdisciplinary Centre of Natural Products, University of Szeged, Eötvös u. 6, 6720 Szeged, Hungary; hohmann.judit@szte.hu

TABLE OF CONTENT

Figure **S1**. ^{1}H NMR spectrum of compound **1** (500 MHz, in $CDCl_3$) 2
Figure **S2**. ^{13}C JMOD NMR spectrum of compound **1** (125 MHz, in $CDCl_3$) 2
Figure **S3**. HSQC spectrum of compound **1** (in $CDCl_3$) 3
Figure **S4**. ^{1}H–^{1}H COSY spectrum of compound **1** (in $CDCl_3$) 3
Figure **S5**. HMBC spectrum of compound **1** (in $CDCl_3$) 4
Figure **S6**. NOESY spectrum of compound **1** (in $CDCl_3$) 4
Figure **S7**. ^{1}H NMR spectrum of compound **3** (500 MHz, in $CDCl_3$) 4
Figure **S8**. ^{13}C JMOD NMR spectrum of compound **3** (125 MHz, in $CDCl_3$) 4
Figure **S9**. ^{1}H NMR spectrum of compound **4** (500 MHz, in $CDCl_3$) 5
Figure **S10**. ^{13}C JMOD NMR spectrum of compound **4** (125 MHz, in $CDCl_3$) 5
Figure **S11**. HSQC spectrum of compound **4** (in $CDCl_3$) 6
Figure **S12**. ^{1}H–^{1}H COSY spectrum of compound **4** (in $CDCl_3$) 6
Figure **S13**. HMBC spectrum of compound **4** (in $CDCl_3$) 7
Figure **S14**. NOESY spectrum of compound **4** (in $CDCl_3$) 7
Figure **S15**. HRMS spectrum of compound **1**. 8
Figure **S16**. HRMS spectrum of compound **4**. 8
Figure **S17.** Chiral chromatographic separation of compound **4**. 9
Figure **S18.** HPLC chromatogram of compound **1**. 9

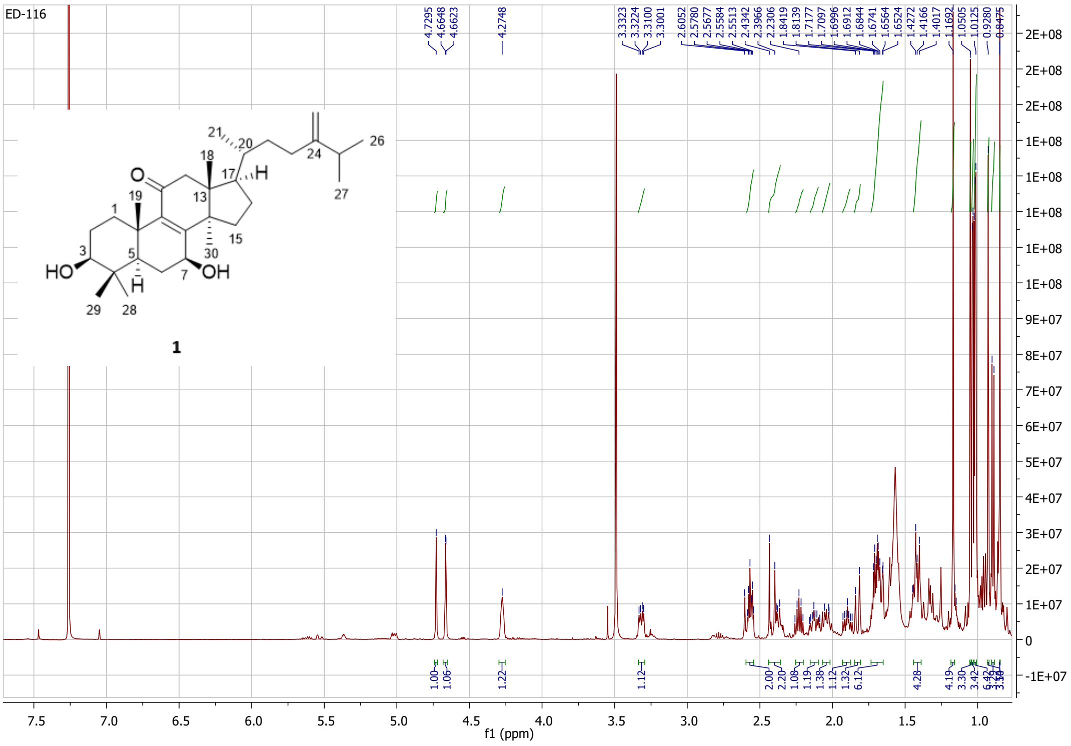

Figure **S1**. ^{1}H NMR spectrum of compound **1** (500 MHz, in $CDCl_3$)

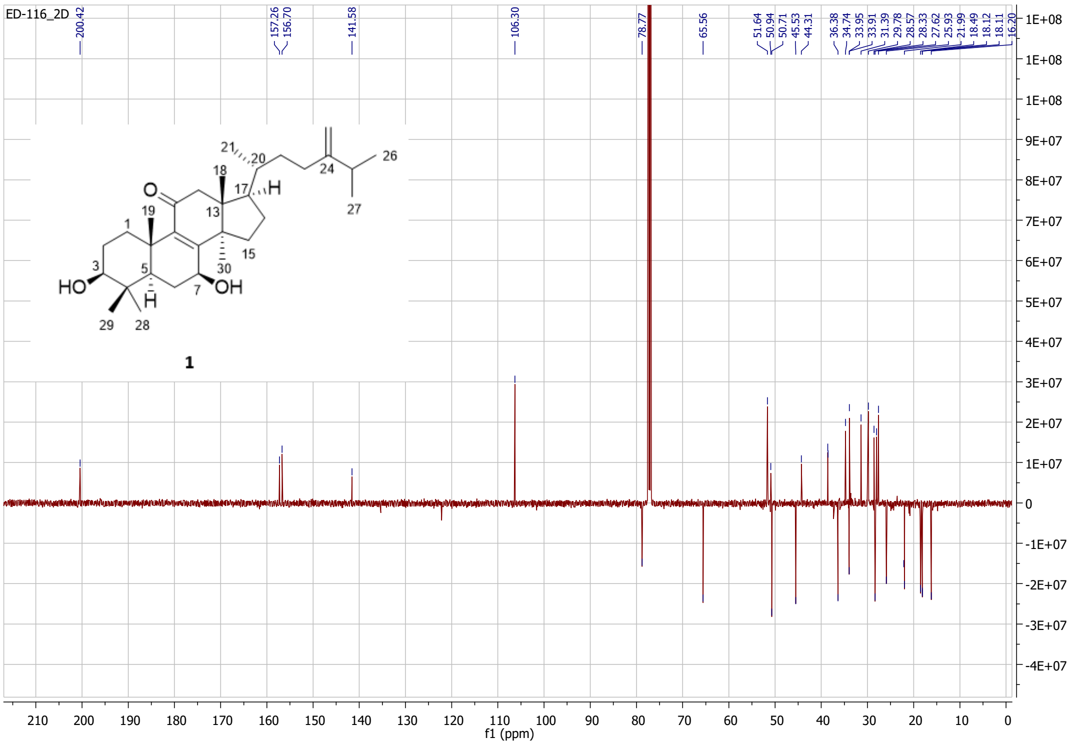

Figure **S2**. ^{13}C JMOD NMR spectrum of compound **1** (125 MHz, in $CDCl_3$)

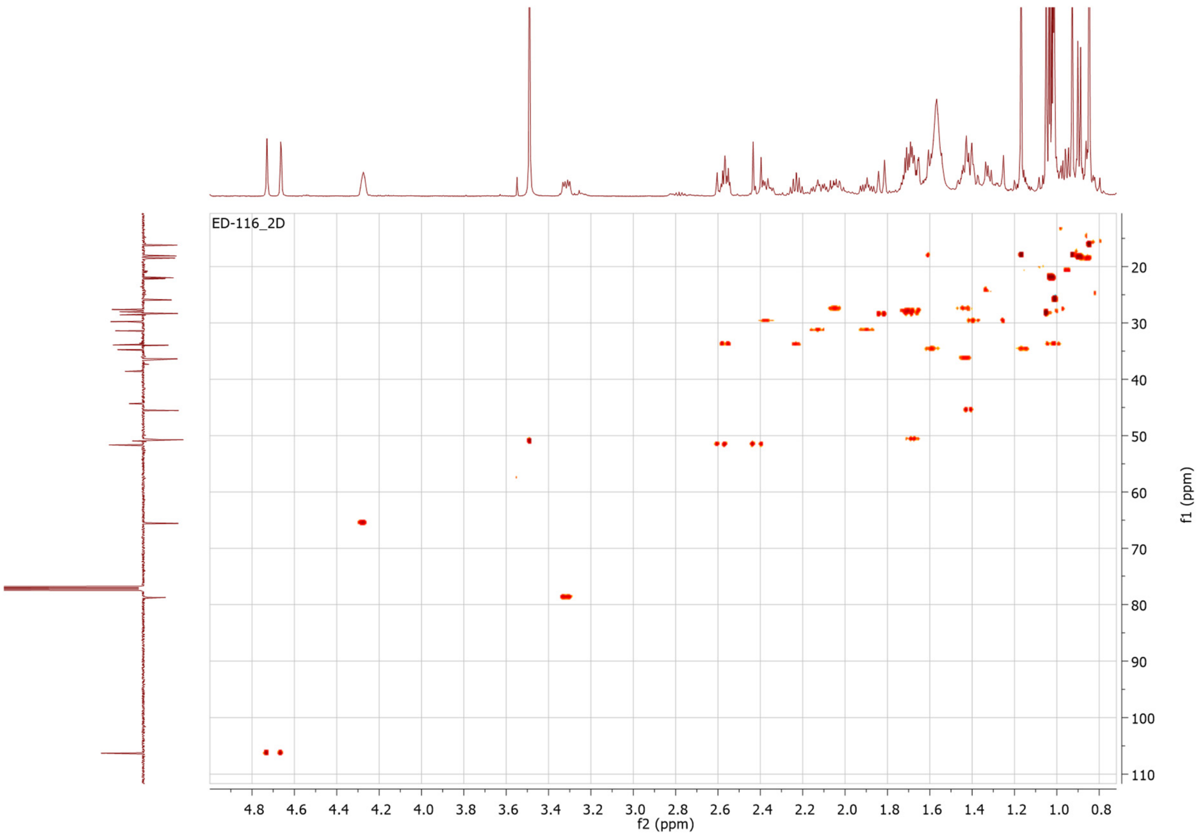

Figure **S3**. HSQC spectrum of compound **1** (in $CDCl_3$)

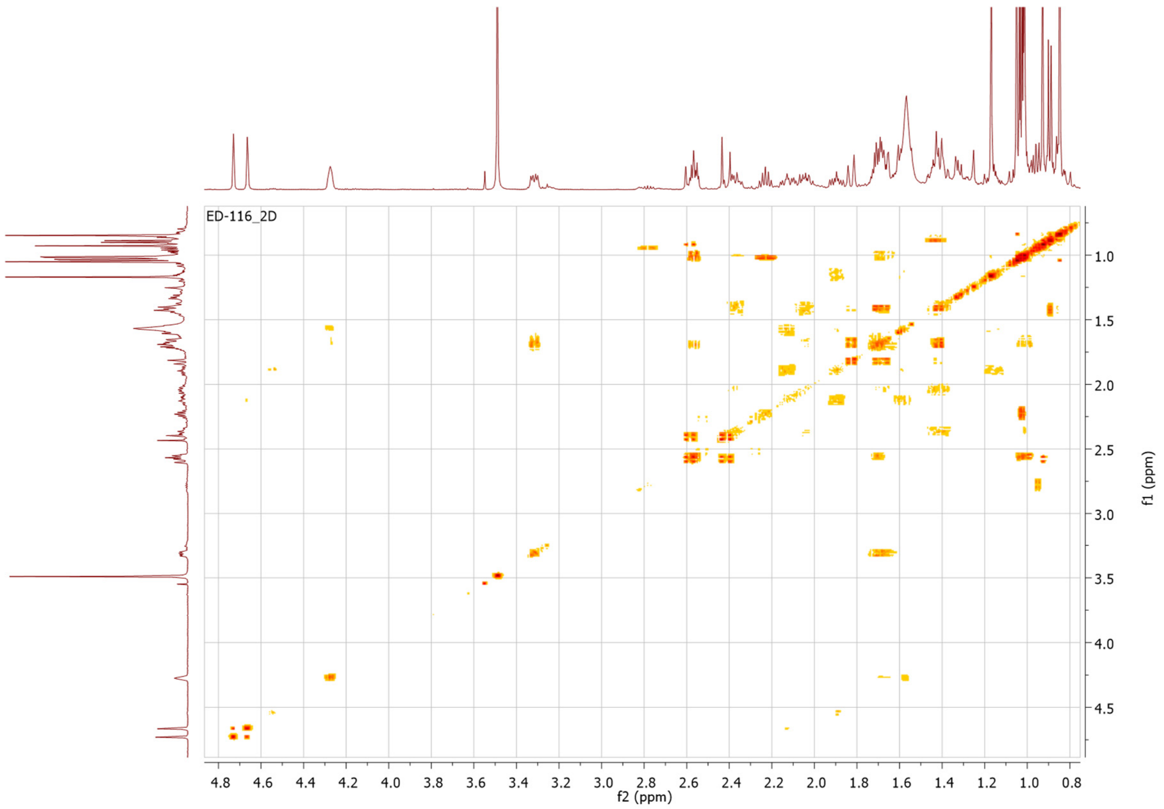

Figure **S4**. ^1H–^1H COSY spectrum of compound **1** (in $CDCl_3$)

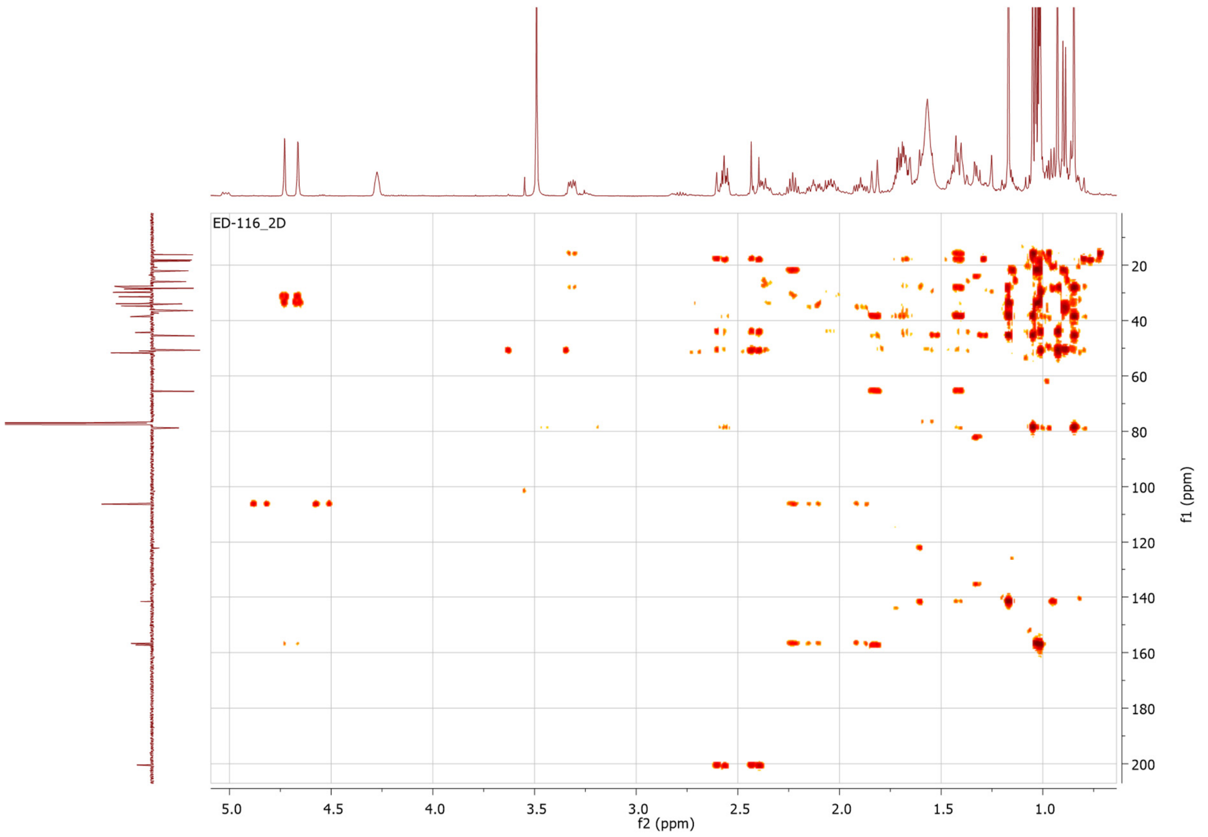

Figure **S5**. HMBC spectrum of compound **1** (in $CDCl_3$)

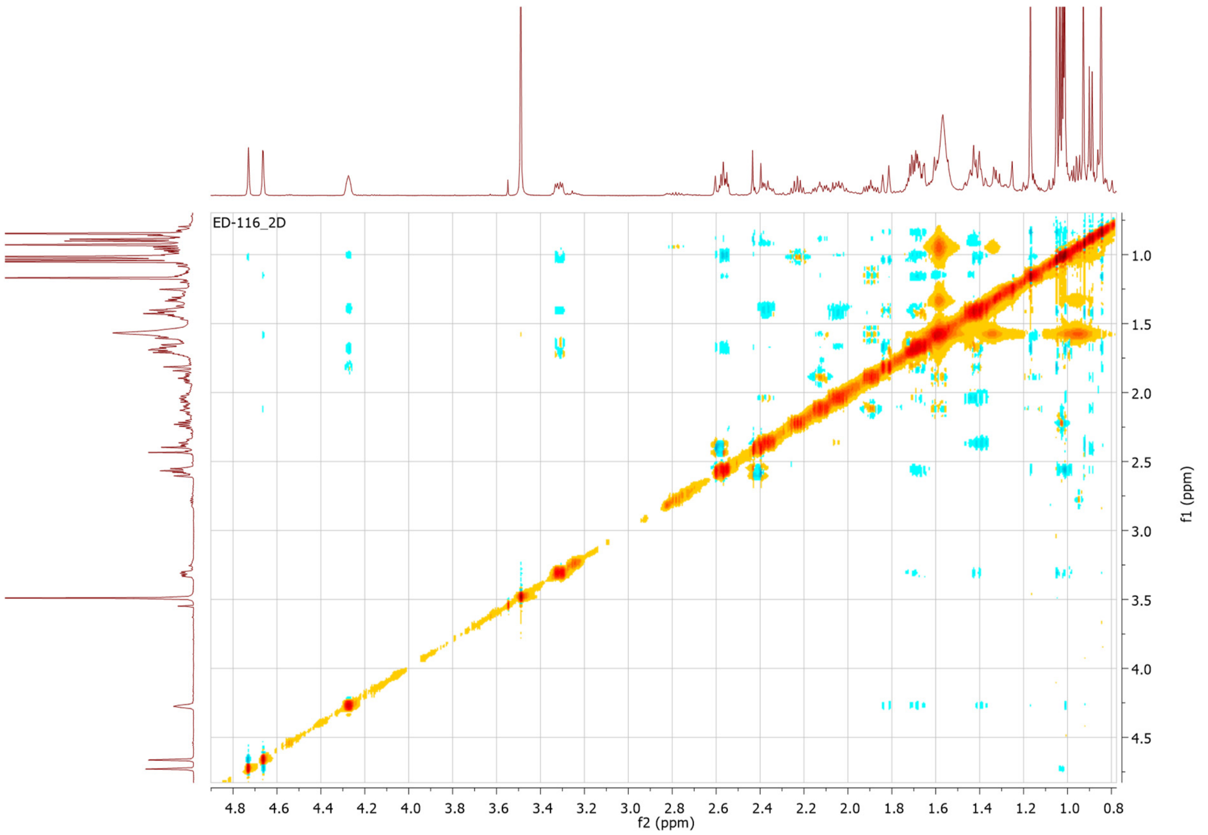

Figure **S6**. NOESY spectrum of compound **1** (in $CDCl_3$)

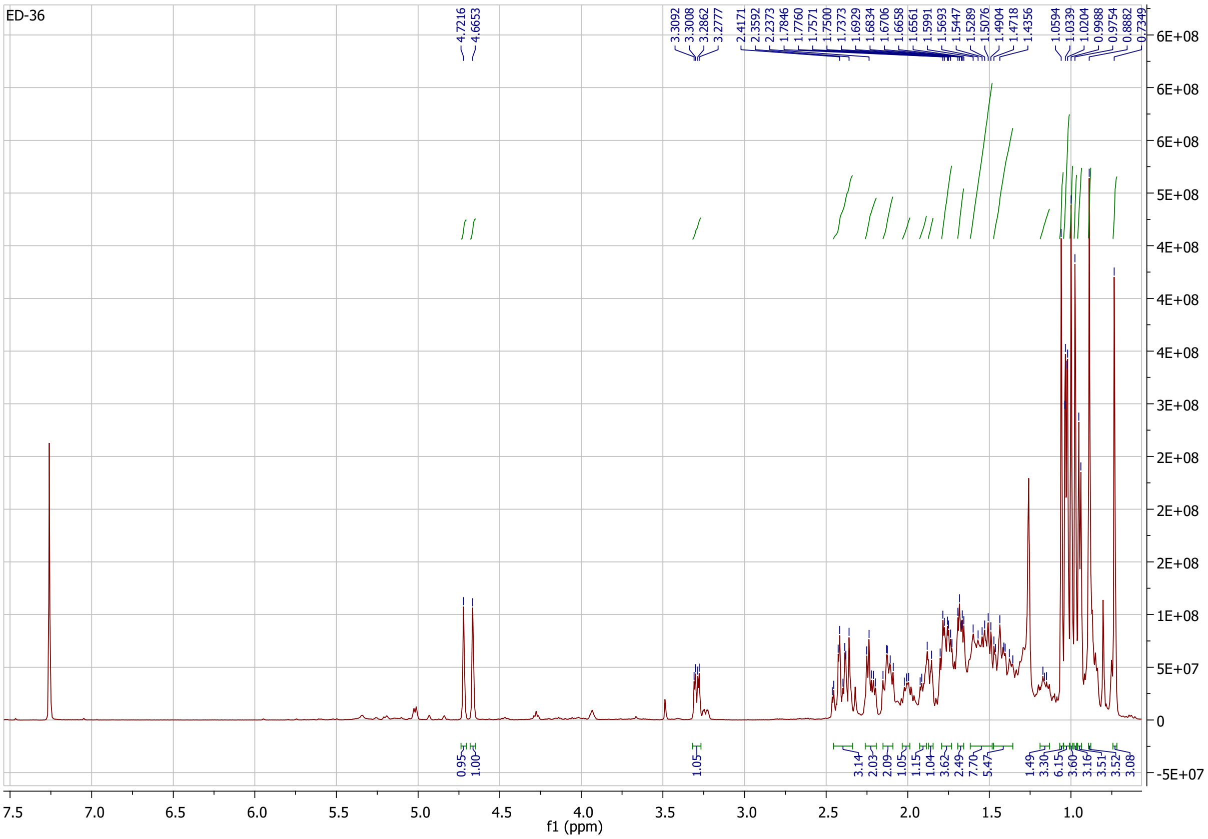

Figure **S7**. ^{1}H NMR spectrum of compound **3** (500 MHz, in $CDCl_3$)

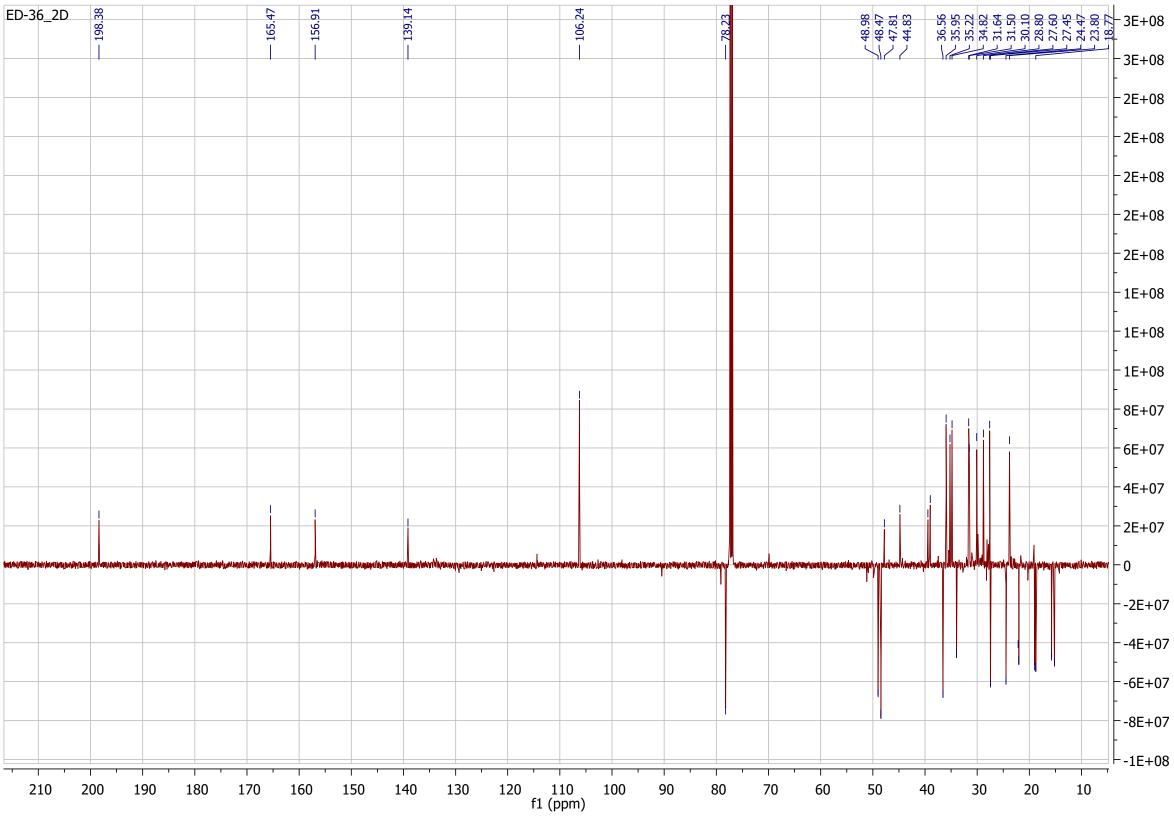

Figure **S8**. ^{13}C JMOD spectrum of compound **3** (500 MHz, in $CDCl_3$)

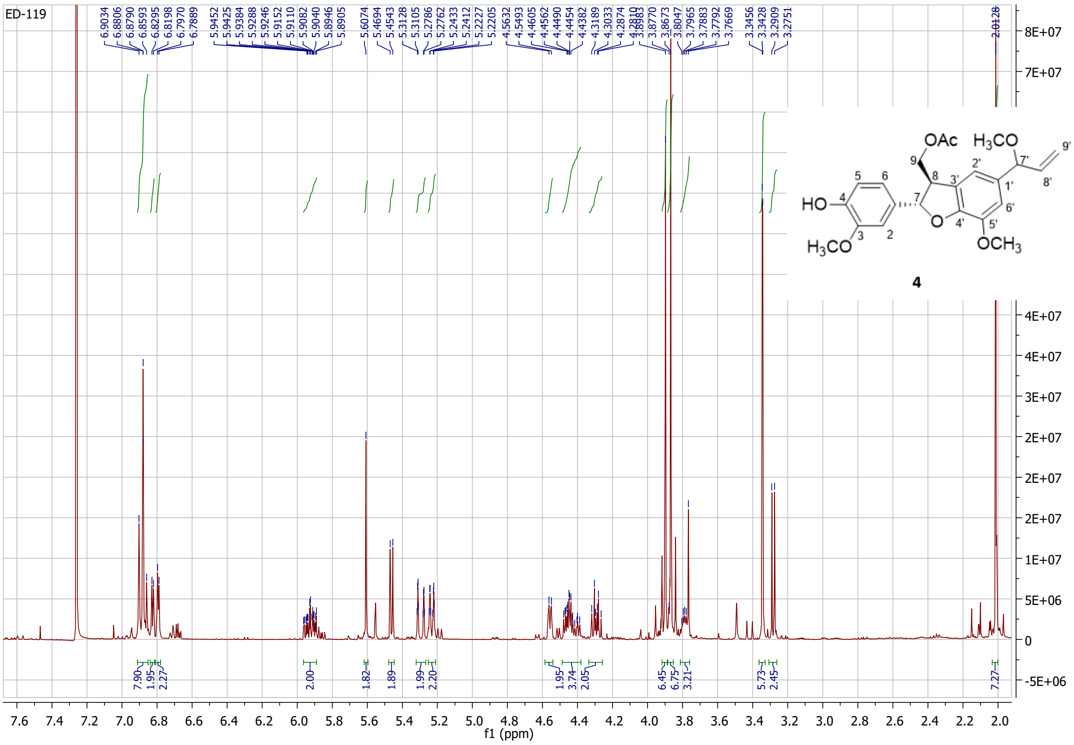

Figure **S9**. ^{1}H NMR spectrum of compound **4** (500 MHz, in $CDCl_3$)

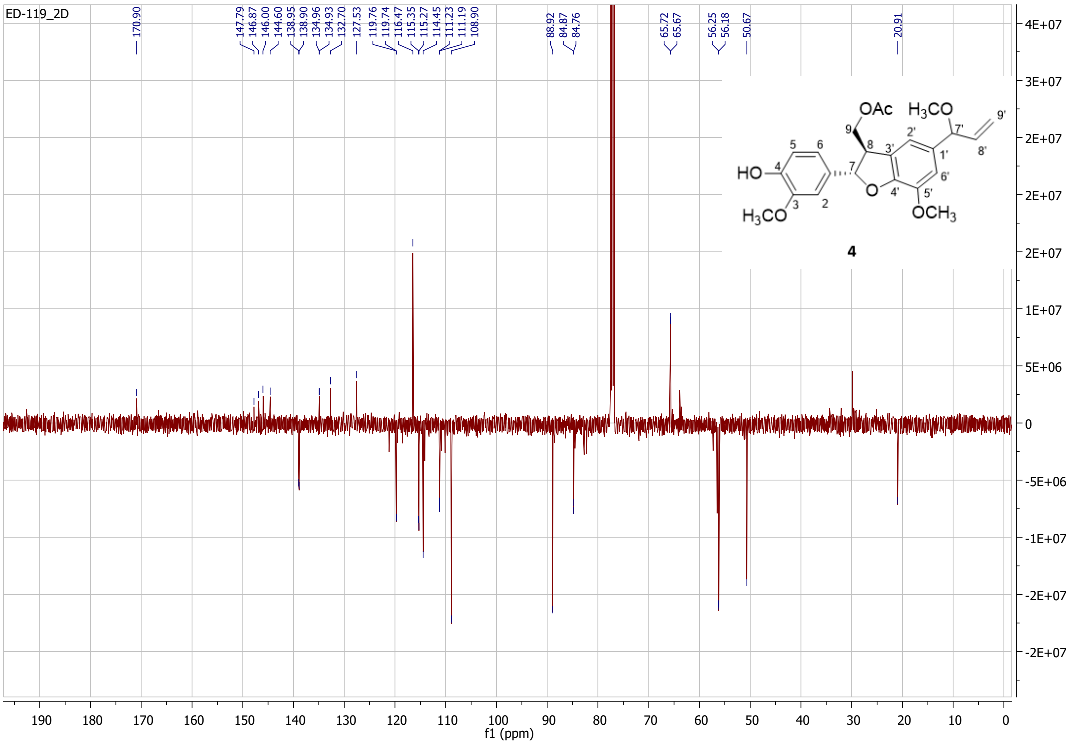

Figure **S10**. ^{13}C JMOD NMR spectrum of compound **4** (125 MHz, in $CDCl_3$)

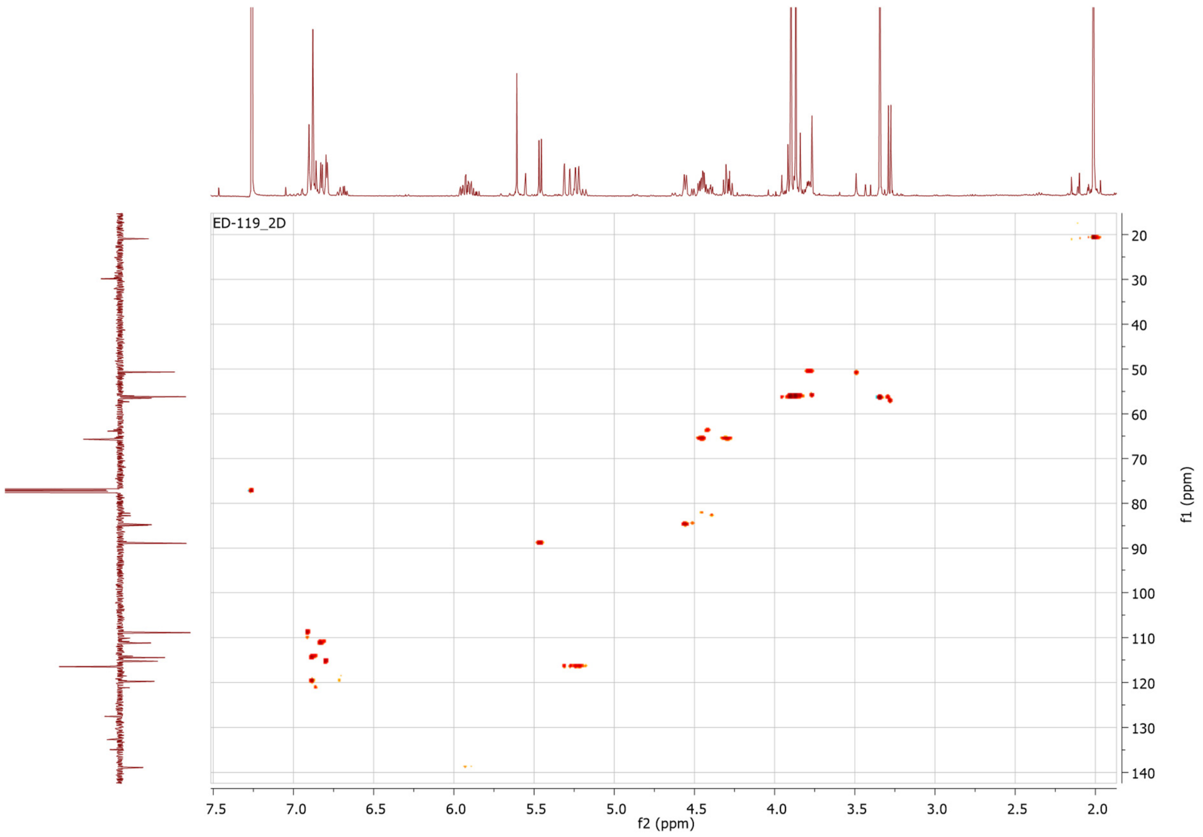

Figure **S11**. HSQC spectrum of compound **4** (in $CDCl_3$)

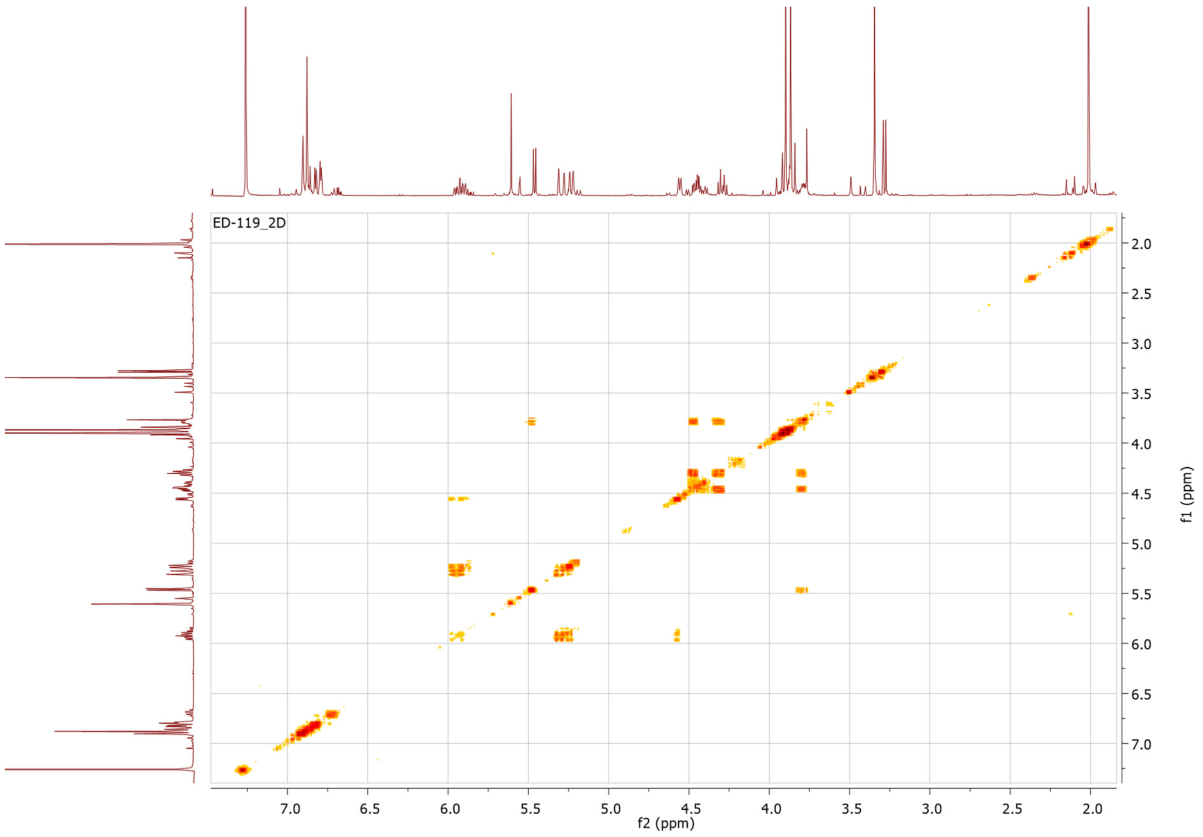

Figure **S12**. ^{1}H–^{1}H COSY spectrum of compound **4** (in $CDCl_3$)

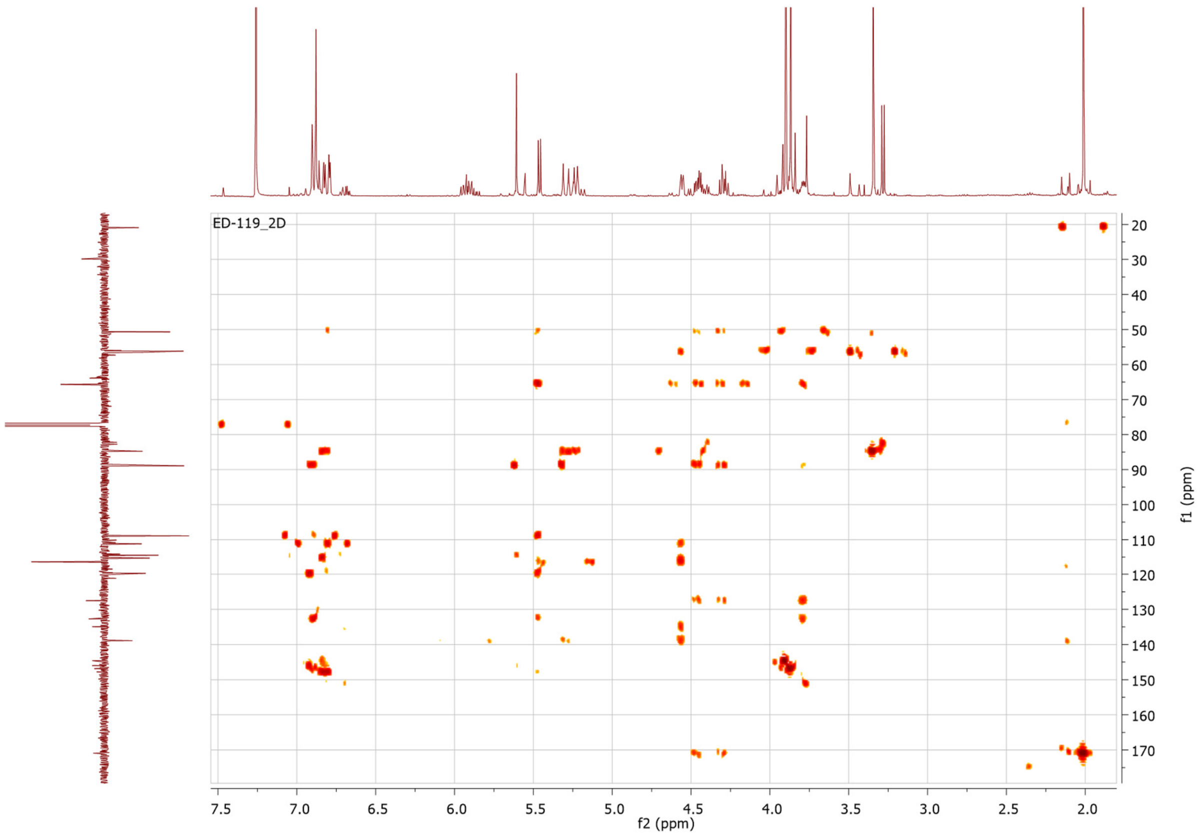

Figure **S13**. HMBC spectrum of compound **4** (in $CDCl_3$)

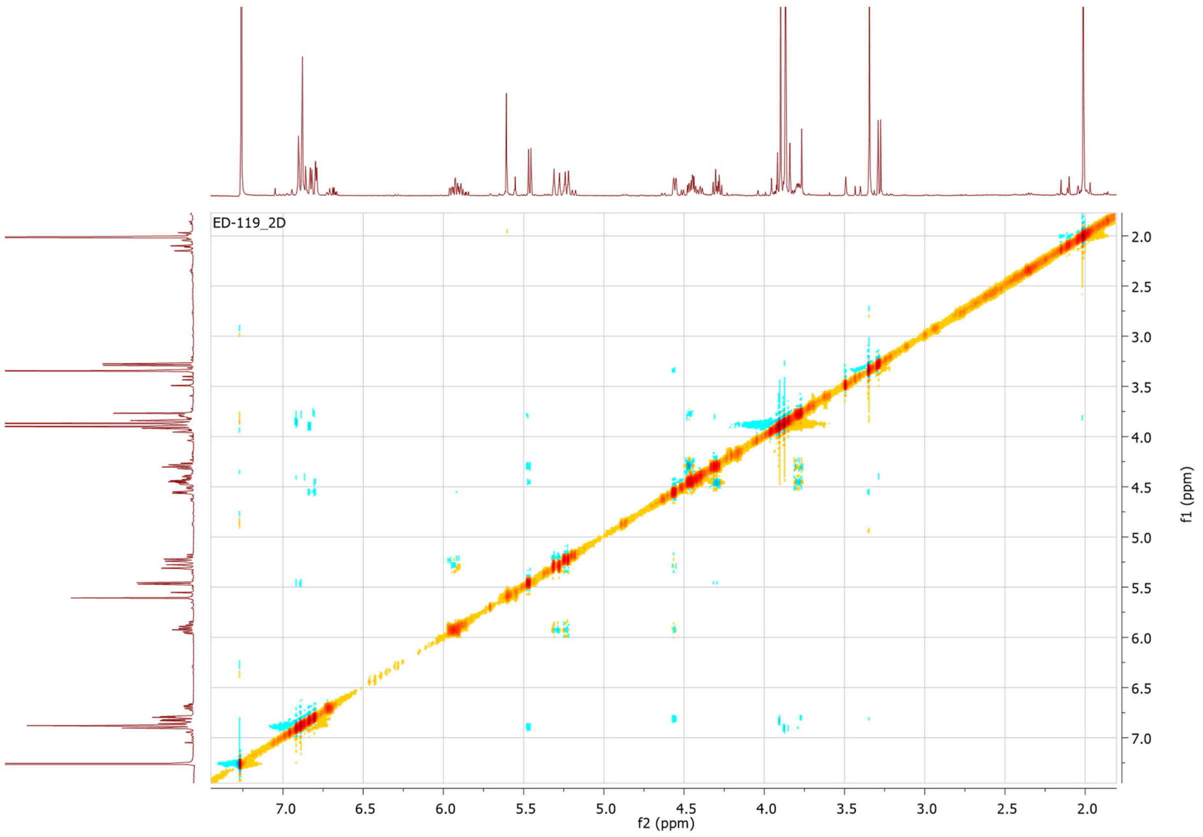

Figure **S14**. NOESY spectrum of compound **4** (in $CDCl_3$)

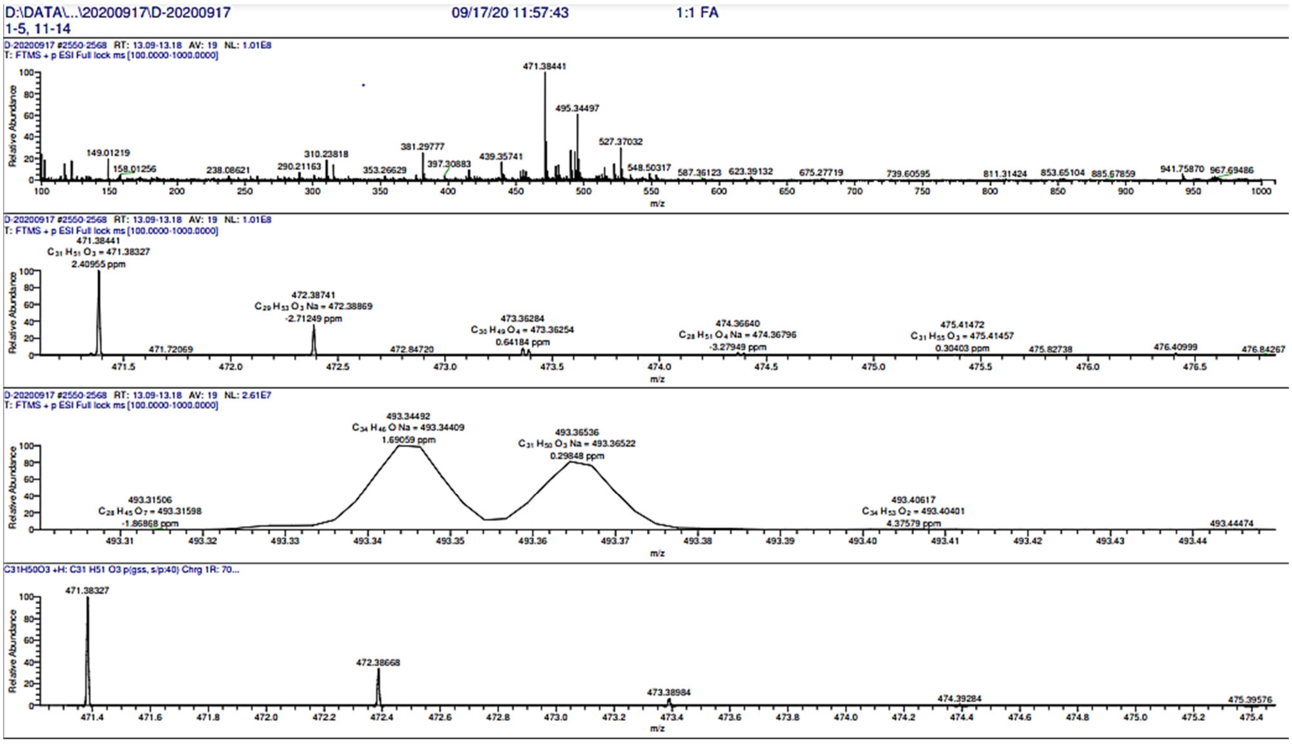

Figure **S15**. HRMS spectrum of compound **1**.

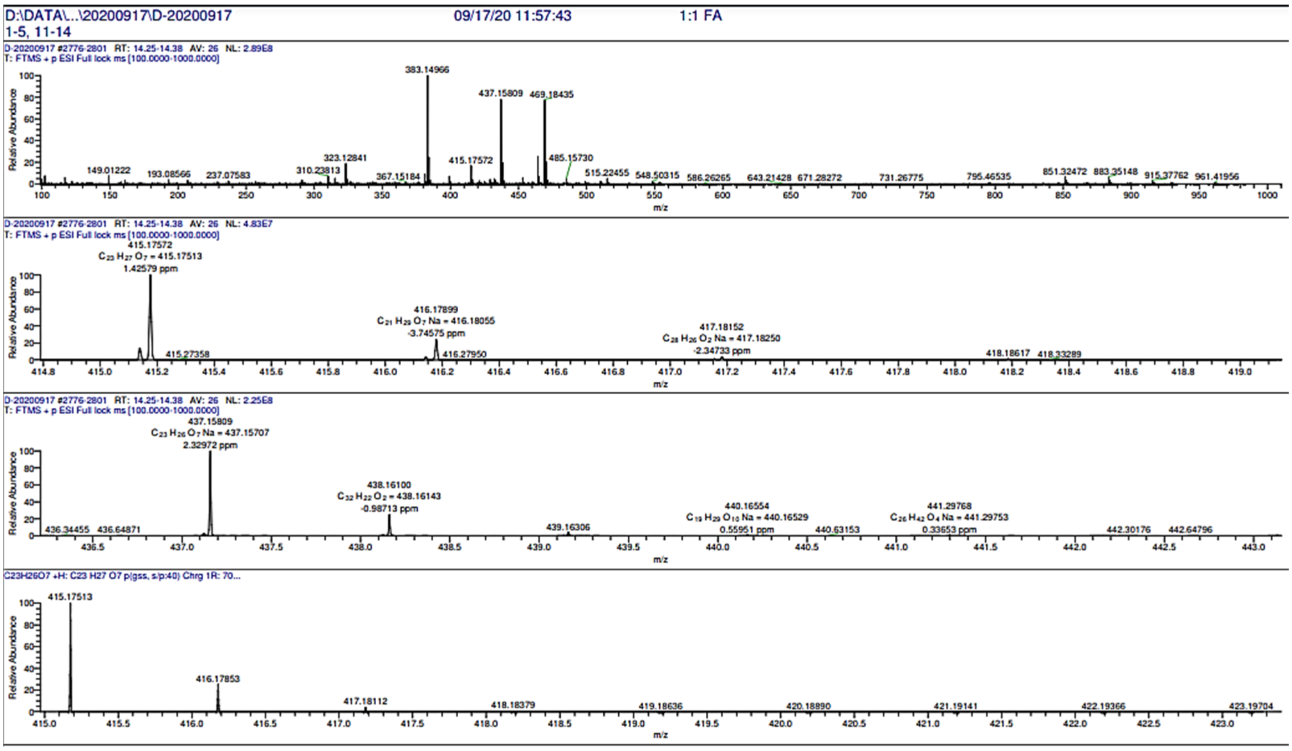

Figure **S16**. HRMS spektrum of compound **4**.

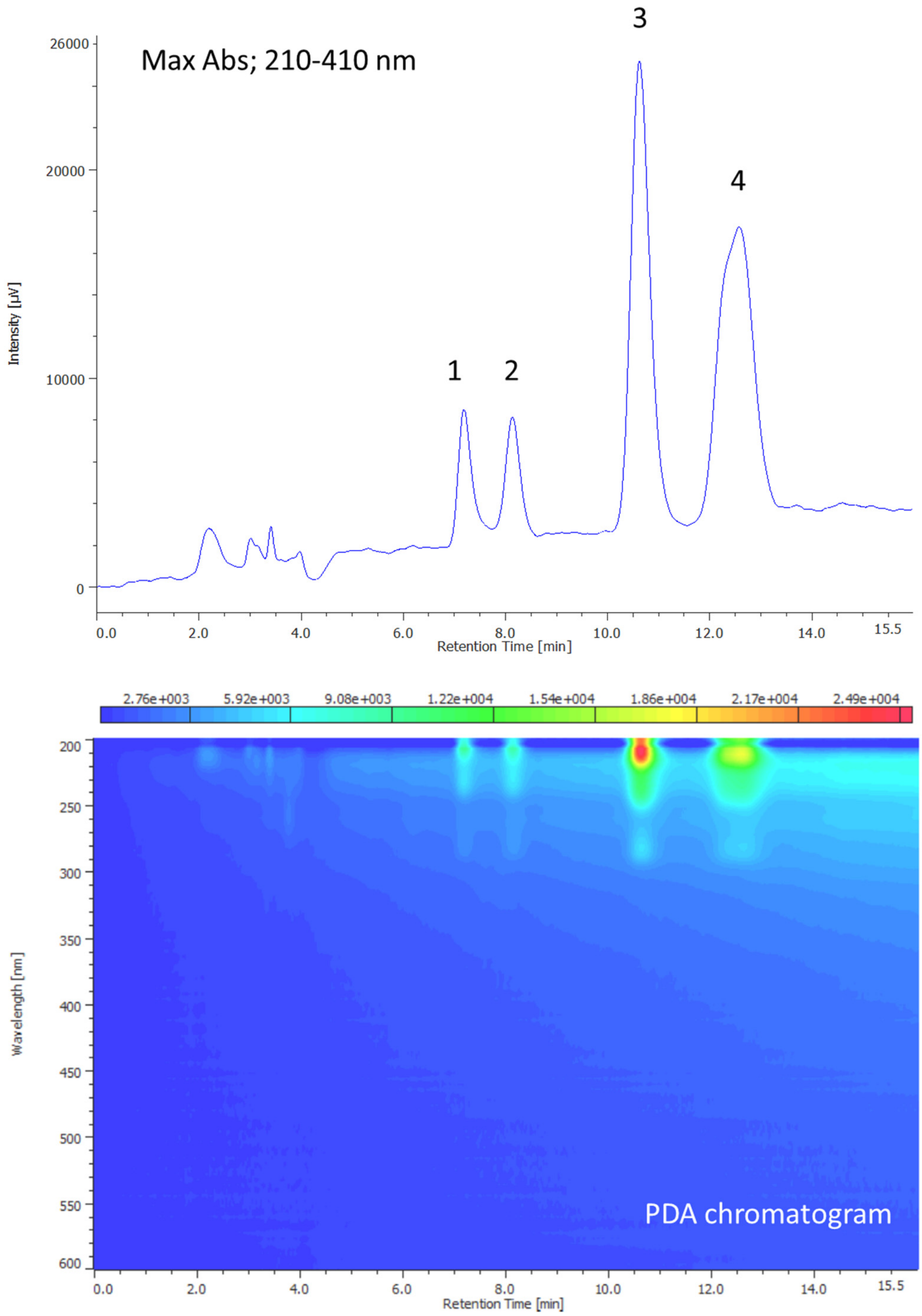

Figure S17. Chiral chromatographic separation of compound **4**. Conditions: chromatographic instrument: Jasco HPLC/SFC instrument (Jasco International Co. Ltd., Hachioji, Tokyo, Japan) equipped with an MD-4015 PDA detector; stationary phase: Phenomenex Lux® 5 µm i-Amylose-1, 250 x 4.6 mm LC column (Phenomenex Inc., Torrance, CA, USA); mobile phase: 1 mL/min flow rate, isocratic elution, cyclohexane (+0.1% TFA) : tetrahydrofuran (+0.1% TFA) – 87 : 13.

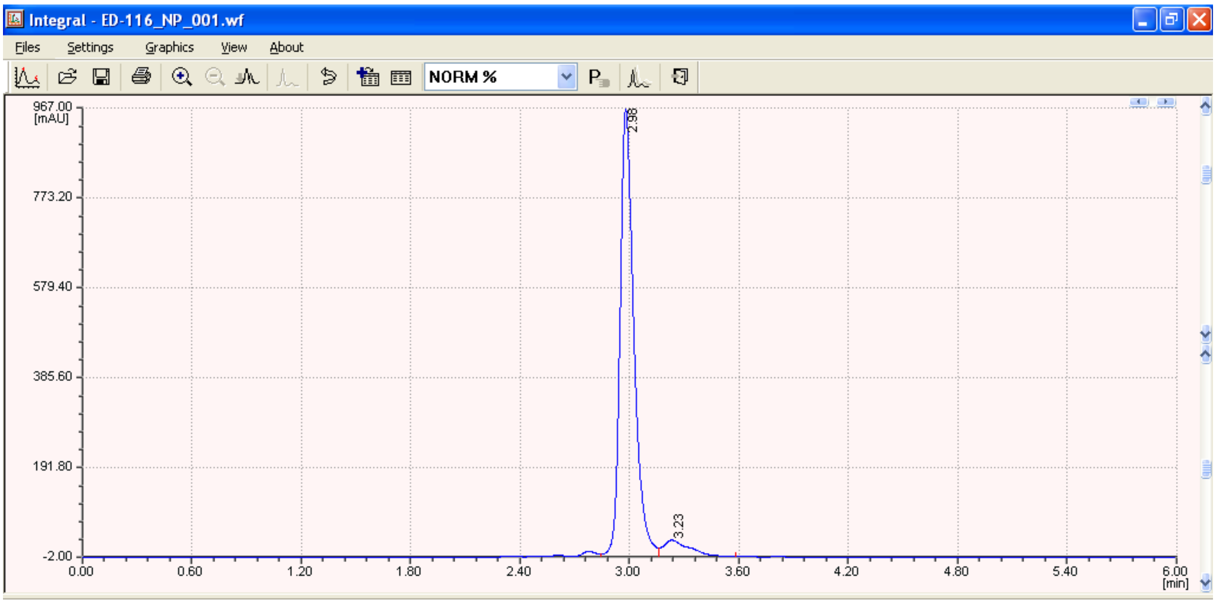

Figure S18. HPLC chromatogram of compound **1**. Conditions: WUFENG HPLC instrument; column: LiChrospher Si 60 (5 µm, 250-4 mm); mobile phase: *n*-Hexane–EtOAc (3:7); flow rate: 1.0 ml/min; RT 2.98 min
